# Assessment of Dioxin and Furan Emission Levels and Management Practices in Addis Ababa, Ethiopia

**DOI:** 10.5696/2156-9614-7.15.85

**Published:** 2017-09-07

**Authors:** Ephrem Sisay Akele, Mekonnen Maschal Tarekegn

**Affiliations:** 1 Addis Ababa City Solid Waste Recycling and Disposal Project office, Addis Ababa, Ethiopia; 2 Environment and Climate Change Management (ECCM) Department, Ethiopian Civil Service University, P.O.Box 26148; Addis Ababa, Ethiopia

**Keywords:** polychlorinated dibenzo dioxin, polychlorinated dibenzo furans, Stockholm Convention, POPs, persistent organic pollutant

## Abstract

**Introduction.:**

An increase in population and related increased demand for health services, expansion of industries, and increasing transportation demands have increased the emission of dioxin and furan persistent organic pollutants (POPs) in Addis Ababa, Ethiopia.

**Objective.:**

This study aimed to identify sources of dioxin and furan emissions, quantify their release into various environmental medias and assess related management practices.

**Methods.:**

The standard United Nations Environmental Programme (UNEP) (2005) toolkit guide and default emission factor were used to identify the main anthropogenic sources and to quantify the amount of released dioxin and furan. Stratified random sampling techniques were applied to assess current management practices.

**Results.:**

Nine main groups of dioxin and furan emission sources were identified. The emission of each source group was calculated by the activity rate data multiplied by an emission factor. The results found that about 138.85 g toxic equivalent(TEQ)/a(TEQ/year)of dioxin and furan were released to air, water, residue (materials remaining as sludge after sewage treatment or in the form of ash after incineration activity) and soil. Waste disposal activities recorded the largest release of dioxin and furan, accounting for 68.30 g TEQ/a of dioxin and furan to water and residue, 34.00 g TEQ/a to air and 0.64 g TEQ/a emitted into soil.

**Conclusions.:**

Several sources of dioxin and furan emission were identified and the present study found that their management is inadequate. Waste disposal services are especially inadequate and generate higher amounts of dioxin and furan gasses. In addition, the organizations that are responsible for the release of dioxin and furan have no awareness of their release and inadequate management practices. The present study points to the need for reformulation of the national legal management framework, adoption of best available technology for disposal services such as incinerators with flue gas management, increasing public and stakeholders' awareness and participation and capacitating the responsible government organizations.

## Introduction

Persistent organic pollutants (POPs) are compounds that resist chemical, biological and photolytic degradation due to their inherent characteristics.^1^ Their low water solubility and high lipid solubility facilitate their bioaccumulation in fatty tissues of living organisms. Many of these compounds are semi-volatile, which enable them to be transported long distances through the atmosphere. “Combined with their overall persistence, POPs are present all over the world, found in every major climatic zone and geographic sector, including deserts; the Arctic and the Antarctic where no major local POP sources exist”.^2^ Once released into the environment, these chemicals (i) remain intact for many years, (ii) become widely distributed throughout the environment as a result of natural processes involving soil, water and, most notably, air, and (iii) accumulate in the fatty tissue of living organisms including humans. They are found at higher concentrations in the food chain, and are toxic to both humans and wildlife.^3^

The presence of POPs in the urban ecosystem increases human health risks. Studies have shown that human exposure to POPs can involve various pathways such as dietary intake, occupational exposure via dust, air, consumer products, dermal absorption and inhalation. POPs may also be transferred from mother to infant via breast milk and umbilical cord blood.^4^,^5^

In Addis Ababa, the population at potential risk of POPs are those living in the vicinity of rivers that are potential discharge points of industrial and residential wastes.^6^ One easily notices the irritating smell of the air in the vicinity of polluted rivers like Akakior Kebena. The rivers in Addis Ababa are used as urban agriculture resources, supplying water to grow vegetables which are supplied to several community markets (known as ‘gulith’) and grocers in different areas of the city and are consumed by a large portion of the population in the city. Other risks come from uncontrolled medical waste combustion, house and vehicle fires, solid waste disposal activities, and iron and steel production activities with limited management practices, and this adversely affects the health of the environment as well as the human population.^3^

Unintentional persistent organic pollutants (unintentional POPs) include polychlorinated dibenzo-pdioxins (PCDDs), polychlorinated dibenzofurans (PCDFs), polychlorinated biphenyls (PCBs) and hexachlorobenzene (HCBs)that are generated unintentionally as a by-product of a given human activity and are addressed by the Stockholm Convention on Persistent Organic Pollutants 2001.^7^ As a result of their unintentional production, unintentional POPS adversely affect the environment and human health without mitigation. The Stockholm Convention called for measures to reduce or eliminate the releases of unintentional production of these pollutants. According to the Stockholm Convention, dioxin and furan emissions are the most significant emissions of concern.

This study aimed to assess the management practices of PCDDs and PCDFs in the capital city of Ethiopia, Addis Ababa, focusing on identification of the main pollutant sources, quantification of emissions, assessment of management practices and available regulatory frameworks.

## Methods

The present study employed a cross-sectional study design. The study was conducted from October 22 to November 21, 2016. Both qualitative and quantitative approaches were used. Primary data were collected using observation, questionnaire and key informant interview. The questionnaire can be found in Supplemental Material 1. Secondary data were then compiled from the internet, government and private organization reports, research articles, United Nations Environmental Programme (UNEP) websites, etc. The collected primary and secondary data were fed into the UNEP tool kit model and Statistical Package for the Social Sciences (SPSS) software.^8^ According to the tool kit guidelines, the total amount of PCDD/PCDFs released per year was calculated and presented in grams of toxic equivalents (TEQ) per year. TEQ is a method of assigning a toxic equivalency factor to report the toxicity-weighted masses of mixtures of dioxins and furans relative to a more potent form (2,3,7,8-Tetrachlorodibenzodioxin (TCDD)), which was assigned the maximum toxicity designation of one.^9^,^10^ In the present study, TEQ was estimated by multiplying the emission factor of each release vector (air, water, land, product (melted iron, for example) and residue) with the amount of feed material processed or product produced (tons or liters per year). The quality of gasses from each category was calculated using grams TEQ/year (g TEQ/a) and the sum of emission of all categories generalized the total amount of PCDD/PCDFs released in Addis Ababa.

### Data Sampling Techniques

Both stratified and random sampling techniques were used. A stratified sampling technique was applied to stratify respondents into groups, then a random sampling technique was used to select an individual organization from each stratum. The dioxin and furan emitting organizations were first stratified; the target samples were then calculated according to total population. Then, samples were further calculated and stratified into private and government sectors according to the ratio of the total number of each population. Representative samples from each private and government organization were taken by its ratio proportion. Finally, organizations were selected randomly from each category to serve as representative samples. Types of organizations interviewed can be found in Supplemental Material 2. Questionnaires were used to collect activity data, as well as other supplementary information. A purposive method of sampling was applied to select samples from organizations with a very small total population and where the ratio of sample proportion was less than one (*Table 1*). The study sample size was determined using a formula as described by Yamane.^11^

**Abbreviations**
*PCDD*: Polychlorinated dibenzodioxin
*PCDF*: Polychlorinated dibenzo furan
*POPs*: Persistent organic pollutants
*TEQ*: Toxic equivalent
*TEQ/a*: Toxic equivalent per year
*UNEP*: United Nations Environmental Programme

**Table 1:**
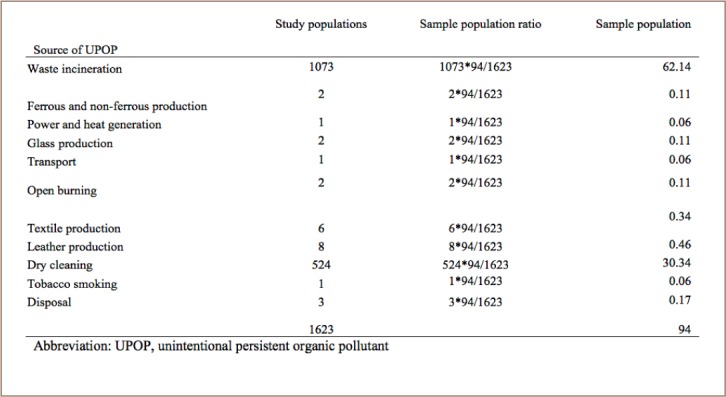
Sampled Population of Respondents

According to the national implementation plan of Ethiopia and UNEP toolkit guide on persistent organic pollutants, the major sources of unintentional POPs include medical waste incineration, ferrous and non-ferrous metal production, power generation and heating, open biomass burning, transport, mineral production, chemical and consumer goods waste generation and disposal. The total number of potential pollution sources considered in the present study was 1623. The representative number of samples that were determined in this study by Yamane scientific formula considered a 90% margin of error.

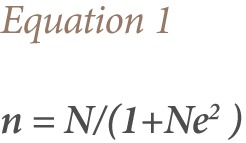
Where,

n: the required sample size
N: the total population
e: allowable error (90%= 0.1)

Hence, n = 1623/(1+1623 (0.1)^2^) = 94

Accordingly, 94 governmental and non-governmental organizations were selected for the present study (*Table 1*).

Finally, the collected data were organized according to the variables, source categories, quantities and management practices of unintentionally produced POPs. Three variables (identification, quantities and management practices) were organized from the respondents' viewpoints. Both qualitative and quantitative methods of data analysis were used to determine unintentional POP management practices. Simple quantitative statistical tools including frequency, percentage, chart and tables were used. Statistical Package for the Social Sciences software (SPSS, Chicago, IL) and the UNEP standard default model for quantification of PCDD/PCDF and Excel program (Microsoft, Redmond, WA) were employed to analyze the data.

### Emission Estimation Methods

Due to the unavailability of specific emission factors, the UNEP (2005) model default emission factors were used to undertake an inventory of annual release of PCDD/PCDF in the study area.^8^

Activity rate, value per year of product manufactured (e.g. steel) or processed fuel (e.g. municipal hazardous waste, coal, diesel fuel, etc.), and annual quantities of material released (e.g. m^3^ of free gas, liter, kilogram or ton of sludge generated, etc.) were used for quantification.

The UNEP toolkit model was used to estimate the annual release of PCDD/PCDF in the study area using Equation 2, as described in the toolkit.^8^


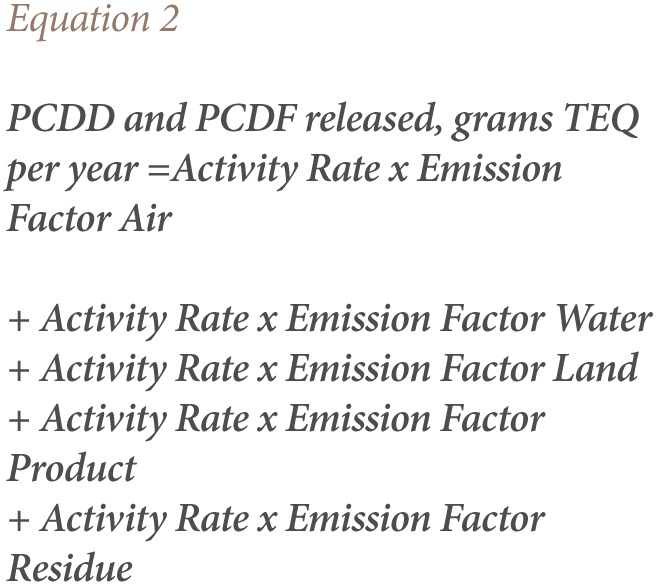


## Results

### Source Groups and Categories of Dioxin and Furan Emissions

Anthropogenic activities such as chemical production, thermal processes, biogenic processes (biological process of sewage disposal/biogenic formation of PCDD/PCDF in sewage sludge) and sewage disposal are major sources of dioxin and furan emissions.

The national implementation plan for the Stockholm Convention prepared in 2015 identified 10 source groups and 28 subcategories of dioxin and furan sources.^6^ In the present study, nine source categories: waste incineration (Group 1), ferrous and non-ferrous metal production (Group 2), heat and power generation (Group 3), production of mineral products (Group 4), transportation (Group 5), open burning (Group 6), production and use of chemicals and consumer goods (Group 7), disposal (Group 8) and miscellaneous (Group 9) were identified and used in the assessment of emission management practices.

### Dioxin and Furan Emissions

Dioxin and furan are organic substances formed by reactions of other chemicals and produced as by-products. The annual release of dioxin and furan was determined based on emission factors and activity rate of pollutants with a method described by the UNEP.^8^

These pollutants were quantified according to their source categories. In 2013, 91.962 g TEQ/a of PCDD/PCDFs were released and dispersed into air and as residue in Addis Ababa.^6^

It was determined that the release amount of dioxin and furan has increased by half compared to the baseline stated in the national implementation plan for Stockholm convention in 2015. According to the present study, 68.30 g TEQ/a of these pollutants were released into waste disposal residue and water. The amount of dioxin and furan released into the air was estimated to be 34.00 g TEQ/a, and only 0.64 g TEQ/a was emitted to soil surfaces.

As shown in Table 2, open burning activities at medical and related institutions, waste incineration and heat and power generation are the biggest contributors of unintentional POPs in Addis Ababa. The results are summarized in Table 2, and the results for each source group were determined by obtaining the activity rate data of the organization and calculating their emissions using the UNEP default emission factor.

**Table 2:**
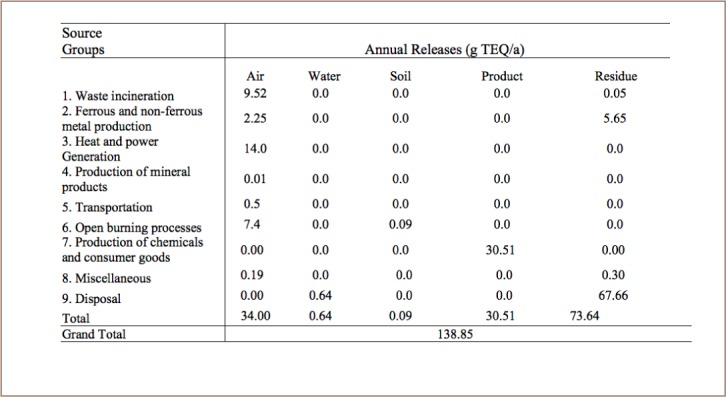
Estimated Annual Dioxin and Furan Release in Addis Ababa

### Release of Dioxin and Furan

Dioxin and furan are emitted from various anthropogenic sources and released to a variety of environmental medias. The amount of dioxin and furan released through different media in the study area was identified and quantified as shown in Table 3.

**Table 3:**
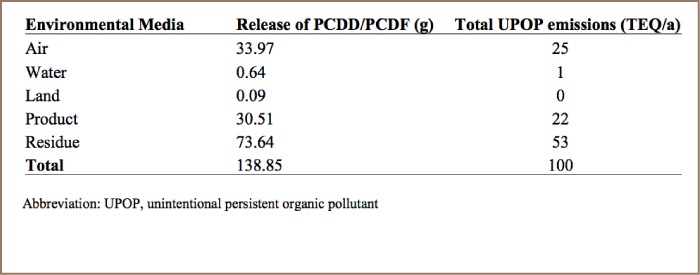
Composition of PCDD/PCDF Emissions in each Vector

### Management Practices of Persistent Organic Pollutants in Addis Ababa

The Stockholm Convention on Persistent Organic Pollutants was created to promote the reduction or elimination of POPs, both intentional and unintentional. Ethiopia is one of the countries that signed and ratified this convention. The findings of the present study on the management practices of unintentionally produced POPs and their sources in Addis Ababa, Ethiopia are discussed below.

#### Medical Waste Incineration

There has been a sharp increase in the quantity of healthcare waste in recent years as a result of rapid population growth and the increasing number of health care facilities.^12^ Health care wastes are either simply disposed of without treatment or incinerated by substandard incinerating facilities. Both disposal methods generate POPs which are then emitted to water, air and soil surfaces. Based on the present study findings, there are 1073 governmental and non-governmental hospitals, clinics, and health care institutions in Addis Ababa.^13^ These health care institutions use substandard incinerators which function under low temperatures and generate higher amounts of dioxin and furan gasses.

As shown in Table 4, only 50% of respondents indicated that they used furnaces for medical waste incineration. Moreover, 100% of respondents reported that these incinerators lack air pollution control systems and medical waste incineration activities at their institutions were not environmentally sound. Finally, about 100% of responses stated that their organizations do not have disposal sites to dispose of the resultant ash. This indicates that the overall management practices of PCDD/PCDF from medical waste incineration are very weak.

**Table 4:**
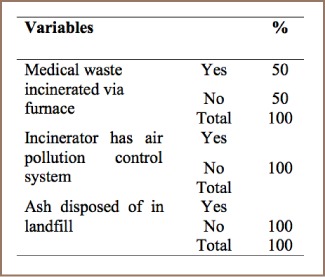
Medical Waste Incineration Management Practices

#### Iron and Steel Production

As summarized in Table 5, all of the respondents indicated that their iron and steel factories used a furnace for heating and melting and cleaning raw materials, but that these furnaces were not equipped with air pollution control systems and PCDD/PCDFs were not properly managed. The lack of air pollution and temperature control systems was confirmed by field observation.

**Table 5:**
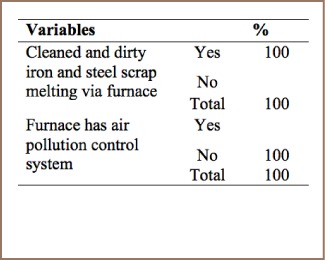
Iron and Steel Production

#### Heat and Power Generation

As shown in Table 6, the three pit stoves, charcoal stove, and other stoves surveyed in the present study lacked combustion control systems, indicating that dioxin and furan are not properly managed.

**Table 6:**
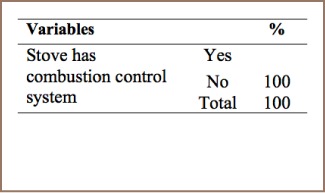
Management Practices for Heating and Cooking with Biomass

#### Glass Production

As shown in Table 7, 100% of respondents stated that their glass factories had a furnace system, but no dust control system. This indicates that PCDD/PCDF emissions from these glass industries are not properly managed.

**Table 7:**
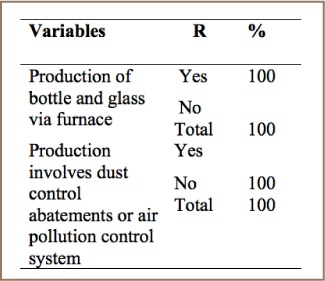
PCDD/PCDF Management Practices in Glass Production

#### Transportation

As shown in Table 8, all respondents from the transportation sector stated that they had no specific management practices such as emission reduction catalysts in their vehicles. This indicates that unintentional POPs emitted from transport vehicles are not properly managed.

**Table 8:**
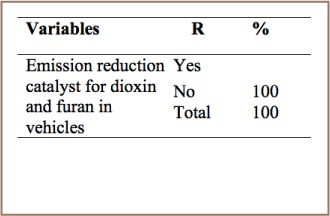
PCDD/PCDF Management Practices in Transportation

#### Open Burning

As shown in Table 9, the pit stoves, charcoal stove and other stoves in the present survey lacked combustion control systems, and PCDD/PCDF emissions from open burning processes were uncontrolled.

**Table 9:**
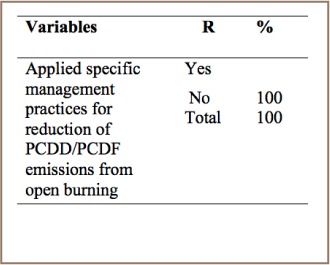
PCDD/PCDF Management Practices in Open Burning

#### Textile Production

As is summarized in Table 10, 75% of respondents stated that waste emitted from chemical and consumer goods industries were simply discharged into the sewage line rather than into a water treatment plant, and only 25% employed water treatment measures.

**Table 10:**
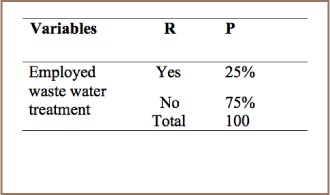
PCDD/PCDF Management Practices in Textile Production

#### Leather Tanneries

As it summarized in Table 11, 99% of respondents reported that tanneries in the study area employed wastewater treatment practices. The remaining 1% of respondents stated that treatment plants are not used and waste is simply discharged into the sewage line, and these findings were confirmed with site visits.

**Table 11:**
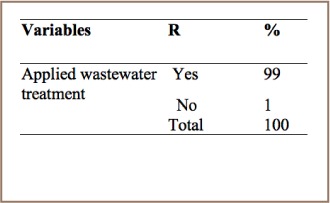
PCDD/PCDF Management Practices in the Leather Industry

#### Dry Cleaning

In the dry cleaning process, PCDD/PCDFs are extracted from textiles and transferred into the cleaning solvent. When the solvent is distilled for recovery and reuse, PCDD/PCDFs are concentrated in distillation residues, which are then disposed of.^14^

As shown in Table 12, 86.66% of respondents revealed that they used chemicals for dry cleaning and disposed of the by-products through the sewage line, and the remaining 13.33% of respondents replied that they collected the distilled by-product into a septic tank. This was confirmed by field observation, which determined that 13% of the respondents utilized septic tanks for disposal of the distilled residue, indicating that PCDD/PCDF management needs improvement in dry cleaning operations.

**Table 12:**
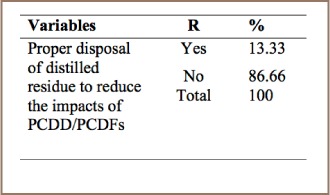
PCDD/PCDF Management Practices in Dry Cleaning

#### Tobacco Smoking

Like any other thermal process, combustion of cigarettes and cigars produces PCDF/PCDDs. As shown in Table 13, tobacco producers have no management practices in place to reduce emissions of unintentional POPs due to tobacco smoking.

**Table 13:**
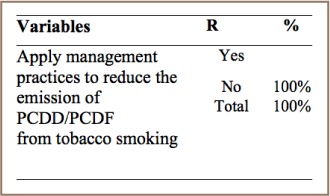
PCDD/PCDF Management Practices in Tobacco Smoking

#### Open Dumping

As indicated in Table 14, 100% of respondents reported that they have no leachate and gas management practices. As reported in previous studies, landfill gases on leachate can escape in an uncontrolled manner without gas collection systems in place. Since solid waste dumping activities are uncontrolled, PCDD/PCDFs are released without mitigation.

**Table 14:**
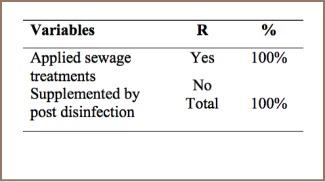
PCDD/PCDF Management Practices in Sewage Treatment

#### Sewage Treatment

PCDD/PCDF concentrations in treated effluent are normally low. However, when chlorine is used to disinfect treated effluent, PCDD/PCDF concentrations can increase; in some cases, by as much as 50-fold.^15^ The present study found that sewage treatment is supplemented by post disinfection activities such as chemical disinfectants. The management practices for sewage treatment facility emissions of PCDD/PCDF are shown in Table 14.

#### Open Water Dumping

As shown in Table 15, about 52.3% of respondents take steps to manage unintentional POPs in the dumping of wastewater.

**Table 15:**
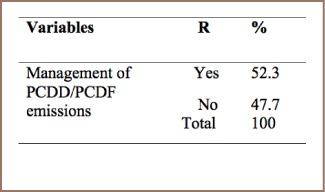
PCDD/PCDF Management Practices in Open Water Dumping

## Discussion

### Institutional Policy and Regulatory Framework for Unintentional Persistent Organic Pollutants

Policy and regulatory frameworks are powerful tools to reduce emissions of pollutants. The United States has taken actions to control and reduce the emission of POPs. The United States Environmental Protection Agency (USEPA) controls and manages the release of dioxins and furans into the air, water and soil through the Clean Air Act and the Clean Water Act.^16^ According to the Ministry of Environment, Forest and Climate Change of Ethiopia, Ethiopia's policy and regulatory framework for POPs has not been put into practice effectively. However, the country has incorporated these issues into various policy and regulatory frameworks such as the Environmental Pollution Control Proclamation No. 300 (2002) and the Environmental Impact Assessment Proclamation No. 299 (2002).^17^,^18^ These regulatory frameworks directly and indirectly influence the management of emissions of POPs and unintentional POPs.

### Main Source Groups and Categories of Dioxin and Furan Emission

According to the UNEP standard toolkit guidelines, the anthropogenic sources of PCDD/PCDF emissions included waste incineration, ferrous and non-ferrous metal production, heat and power generation, production of mineral products, transportation, open burning, production of chemicals and consumer goods, and disposal and other miscellaneous activities.^8^ These emission sources were also included in the Stockholm Convention on the reduction of unintentional POPs. According to the Federal Democratic Republic of Ethiopia's national implementation plan, about ten source groups and 28 subcategories were identified as sources of dioxin and furan emissions.^6^ In the national implementation plan report information, about 10 source groups were identified in Addis Ababa.

### Dioxin and Furan Emissions

Dioxin and furan are organic substances formed by chemical reactions of certain processes or activities. They are very toxic to the environment as well as human health. According to the UNEP, quantification of dioxin and furan emissions is a difficult task globally.^7^ The first nationwide PCDD/PCDF release inventory was undertaken in 2013 and the inventory found that 91.96 g TEQ/a of dioxin and furan was released due to burning, and 56.172 g TEQ/a was released due to medical waste incineration. Third, production of chemicals and consumer goods such as textiles, leather, and pulp production released 28 g TEQ/a of PCDD/PCDF to air and product. Emissions from the others sources were insignificant.

The results of the present study varied in value and level compared to this previous national inventory. The present study determined the quantification from nine source groups and 15 source categories. In the study area, waste disposal activities accounted for a 75.220 g TEQ/a release of PCDD/PCDF to residue and water. This indicates that the rate of waste generation has increased over open burning since the first national inventory of PCDD/PCDF was undertaken in 2013.

Production of chemicals and consumer goods such as leather and textile production are the second biggest source of PCDD/PCDF releases and accounted for 30.51 g TEQ per product. This indicates that these industries have expanded production and emissions in the study area within the last decade. The third largest anthropogenic source was heat and power generation, with a release of 14.03 g TEQ/a of PCDD/PCDF to air. However, power generation activities ranked fifth in PCDD/PCDF release due to high demand for power. As summarized in Table 2, other source groups such as medical waste incineration, ferrous and non-ferrous metal production, open burning, transport, and miscellaneous activities such as dry cleaning and tobacco smoking also make significant contributions to dioxin and furan emissions.

### Release of Dioxin and Furan to Different Environmental Media

As documented in previous studies and detailed in the Stockholm Convention, dioxin and furan emissions are released into five environmental vectors: air, water, soil, product and residue. Medical waste incineration, iron and steel production, household heating, production of mineral products such as glass, transportation, and open burning release dioxin and furan emissions via the air.^7^ Production of chemicals and consumer goods such as leather and textile factories release PCDD/PCDFs into product media. PCDD/PCDFs are emitted to residue through open dumping, sewage and open dumping of water from dry cleaning activities.

### Current Dioxin and Furan Management Practices in Addis Ababa

In developing countries, the quantity of health care waste has sharply risen in recent years due to rapid population growth and increasing demand for health services. Despite large investments in expanding public and private health care facilities in most developing nations, healthcare wastes are usually disposed of in the environment without any treatment.^12^,^19^ There are 1073 governmental and non-governmental hospitals, clinics, and health care institutions in Addis Ababa.^13^ These health care institutions generate medical waste and practice uncontrolled incineration.

There are two iron and steel factories in Addis Ababa. These factories have intensive use of raw materials such as ore, pellets, scraps, coal, lime, and in some cases also heavy oil and plastics and additives. They are highly energy intensive with more than half of the mass input becoming output in the form of release to air, soil, waste or by-products.^5^ The factories use dirty and clean raw materials to produce iron and steel without air pollution control systems on the furnaces during the melting process, resulting in high amounts of PCDD/PCDF released to the air.

Like many developing countries, heating and cooking with biomass in residential households are common practices in Addis Ababa. None of the biomass stoves used for heating and cooking were found to have combustion control systems.

Furnaces used for glass manufacturing may be continuously or intermittently operated and typical fuels are oil and gas.^3^ The two glass factories in Addis Ababa use raw materials such as sand, limestone, dolomite, soda, and in some cases, recycled glass. In addition, a wide range of other materials may be used to achieve desired properties such as color, clarity and purification. These factories do not practice dust control abatement to reduce the emission of PCDD/PCDFs.

Levels of PCCD/PCDF in exhaust gasses from vehicles depend on many factors, including the type of engine, maintenance condition and age, emission reduction technologies (catalyst), type and quality of fuel, driving conditions, current condition, etc.^3^ Most of the cars in Addis Ababa are old and have inadequate emission control technologies and therefore release PCDD/PCDFs into the air.

As has been reported in previous studies, PCDD/PCDFs have been detected in distillation residues from dry cleaning. In the dry cleaning process, PCDD/PCDFs are extracted from the textiles and transferred into the cleaning solvent.^14^ PCDD/PCDFs are concentrated in the distillation residues, which are then disposed of. In the study area, it was determined that the chemicals distilled from residue and distillate of dry cleaning is simply dumped into surface water.

In contrast to sanitary landfills, open waste disposal pits, dumps and waste piles have no engineered design and structure to control contamination and are largely unregulated and uncontrolled. Rain and other water runs through the waste in landfills, generating contaminated leachate runoff. Where no leachate collection systems are installed, landfill gasses on leachate escape from the dump in an uncontrolled manner with higher amounts of PCDD/PCDF. As previously reported, landfill leachate or seepage and nearby soils have higher concentrations of dioxin and furan pollutants.^20^ Hence, lack of waste management systems results in higher emission of these gasses.

When chlorine is used to disinfect treated effluent, PCDD/PCDF concentrations can increase, in some cases, by as much as 50-fold.^15^ The present study found that chlorinated compounds are used to disinfect treated sewage effluent, but dioxin and furan is then discharged into nearby water bodies with the effluent.

### Policy and Regulatory Framework for Dioxin and Furan

According to the Clean Air Act, the USEPA seeks to identify the major industrial sources of toxic air emission and set regulations using technology and performance-based approaches to reducing toxic emissions. Industries are required to achieve the maximum control of hazardous air pollutants, including dioxins and furans.^16^ The Clean Water Act controls and manages the release of dioxins to water through a combination of risk-and technology-based tools. The present study found that there are no locally adapted regulatory tools relevant to dioxin and furan emission control in Addis Ababa, even though several legislative policies are in place. The Environmental Pollution Control Proclamation No. 300 (2002) and the Pesticide Registration and Control Council of State Special Decree No. 20 (1990) are among the most important legislative actions for regulating POPs in Ethiopia.^17^,^18^ Due to weak institutional management practices, lack of implementation guidelines and standards and low levels of stakeholder collaboration, these regulations have not been satisfactorily implemented and enforced.

## Conclusions

The Stockholm Convention on Persistent Organic Pollutants states that each party to the convention should reduce emissions of unintentional POPs at their source, or if possible, eliminate the emission of PCDD/PCDFs.^3^ The present study focused on the management of unintentional POPs in Addis Ababa, Ethiopia. The results revealed that few measures are in place to regulate or control emissions in the study area. The environmental policies and strategies outlined in the Stockholm Convention have been accepted and approved. However, specific rules and regulations for the direct management of POPs do not yet exist at the city and national level. This lack of governance may lead to increased emissions of PCDD/PCDFs from year to year. The following recommendations are suggested to improve the management of unintentional POPs and to reduce their emissions in Addis Ababa.

### Technological Improvement

The majority of unintentional POPs are released as a result of poor technology, therefore it is recommended that the best available technologies and best environmental practices in power generation and waste incineration be adopted to reduce or eliminate releases of PCDD/PCDFs through detailed assessment of individual industries and their options for unintentional POPs management.

### Capacity Building

Capacity development, awareness raising and participation of public and stakeholders are essential to improve the management practices of dioxin and furan in Addis Ababa. Establishment of awareness creation platforms such web data bases and working organizational structures are recommended.

### Regulatory Framework

Preparation and implementation of relevant regulations, standards and guidelines are recommended to regulate and reduce unintentional POP emissions. The city administration should work in collaboration with the federal government on formulating the regulatory framework related to the signed Stockholm Convention on Persistent Organic Pollutants.
