# Supplementary material for: Assessment of Dioxin and Furan Emission Levels and Management Practices in Addis Ababa, Ethiopia
Source: J Health Pollut. 2017 Sep 7;7(15):85–94. doi: 10.5696/2156-9614-7.15.85 (PMC6236542; doi:10.5696/2156-9614-7.15.85)
Supplement: Supplementary file 1 [file Tarakegn_Supplemental_Material_1.docx]

**Supplemental Material 1**

**Data Collection Tool I: Questionnaire for Governmental and Non-Governmental Organizations (Activity Data Collection)**

**Reference Year:** November, 2016

**Requested by:** EphremSisayAkele (Tel: +251-913 82 18 51) &

Mekonnen Maschal Tarekegn (Tel: +251911879718)

*Email:* [*ephremsisay35@yahoo.com*](mailto:ephremsisay35@yahoo.com)*&*[*maschalm12@gmail.com*](mailto:maschalm12@gmail.com)

**Dear Respondent,**

The main objectives of this questionnaire are to obtain data for identifying, characterizing, quantifying and prioritizing sources of unintentionally produced persistence organic pollutantsandto evaluate the management practices and source categories (UPOP contributors) in Addis Ababa. The findings will be applicable as an input to policy makers on the safety of the environment and public health of localities. Keep in mind that the quality of the research work is highly dependent on the accuracy of your responses. Therefore, we need your genuine responses as much as possible. The researcher would like to thank you in advance for your commitment and truthful responses.

**Questionnaire 1:Group_1Waste Incineration**

**Part A: incineration**

| Type of Plant | Medical Waste Incinerator |  |
| --- | --- | --- |
| Name of Plant |  |  |
| Location(City/Province) |  |  |
| Address􀀃 |  |  |
| Contact  (Name, position) |  |  |
| Phone numbers and email |  |  |
| Number of Furnaces  Type of Operation | Batch(*e.g.*,100 kg per batch)  Semi continuous(*e.g.*,8hoursperday)  Continuous(24hours per day) |  |
| Annual Operational/Capacity  (per unit) | t/h (tons per hour) |  |
|  | h/d (hours per day) |  |
|  | d/w(days per week) |  |
|  | t/d (tons per day) |  |
|  | d/a (days per year) |  |
|  | h/a(hours per year) |  |
| Annual Operation/Capacity  (total) | t/a(tonsperyear) |  |
|  | t/h (tons per hour) |  |
|  | h/d(hours per day) |  |
|  | d/w(days perweek) |  |
|  | t/d(tons per day) |  |
|  | d/a(days per year) |  |
|  | h/a (hours per year) |  |
|  | t/a (tons per year) |  |
| Type of Furnace | Mass burn waterwall (grate) |  |
|  | Fluidized bed |  |
|  | Stoker |  |
|  | Rotary kiln |  |
|  | Other (pleasespecify) |  |
| Temperature in Furnace | Main furnace(°C) |  |
|  | Afterburner/second chamber(°C) |  |
| Type of Air Pollution Control | System(APCS) |  |
|  | Electrostatic precipitator |  |
|  | Cyclone |  |
|  | Bag filter |  |
|  | Wet scrubber |  |
|  | Dry scrubber |  |
|  | Lime injection |  |
|  | Na OH/alkali injection |  |
|  | Active carbon/coke injection |  |
|  | Active carbon filter |  |
|  | Catalytic converter(SCR) |  |
|  | Induced or forced draft fan |  |
|  | Other (please specify) |  |
|  | None |  |
| Heat Recovery System |  |  |
| Temperature of Gases | At entry to APCS (°C) | At exit from APCS (°C) __________ |
| Flux of Exit Gases | At exit from APCS(°C) |  |

**Part B: Residue/Bottom Ash Management**

| **Residue** | **Quantity** | **Management Methods** | **Residue Disposal** | |
| --- | --- | --- | --- | --- |
|  |  |  | **Disposal site** | **Quantity (t/a)** |
| Generation of Bottom Ashes | t/a | Recirculation | Landfill |  |
| Generation of Fly Ashes | t/a | Recirculation | Landfill |  |
| Generation of waste water | t/a | Disposal | Leachate treatment |  |
| Generation of Slides | t/a | Recirculation | Landfill |  |

**Questionnaire 2: Group_2 Ferrous and Non-Ferrous Metal Production**

**Part A: Ferrous and Non-Ferrous Metal Production**

| Name of Plant |  | |
| --- | --- | --- |
| Location(City/Province) |  | |
| Address |  | |
| Contact  (Name, position, phone numbers, email) |  | |
| Type of Plant | Sinter |  |
|  | Coke |  |
|  | Iron and/or steel |  |
|  | Foundry |  |
|  | Copper |  |
|  | Aluminum |  |
|  | Lead |  |
|  | Zinc |  |
|  | Brass/Bronze |  |
|  | Magnesium |  |
|  | Other non-ferrous metal  Shredder |  |
| Number of Furnaces  Type of Operation | Batch(*e.g.*,100 kg per batch) [ ]  Semi continuous(*e.g.*,8hours per day) [ ]  Continuous(24hoursper day) [ ] |  |
| Annual Operational/Capacity  (per Unit) | t/h(tons per hour) |  |
|  | h/d(hours per day) |  |
|  | d/w(days per week) |  |
|  | t/d (tons per day) |  |
|  | d/a (days per year) |  |
|  | h/a (hours per year) |  |
| Annual Operation/Capacity  (total) | t/a(tons per year) |  |
|  | t/h (tons per hour) |  |
|  | h/d (hours per day) |  |
|  | d/w (days per week) |  |
|  | t/d (tons per day) |  |
|  | d/a (days per year) |  |
|  | h/a (hours per year) |  |
|  | t/a (tons per year) |  |
| Type of Furnace | Mass burn water wall (grate) |  |
|  | Fluidized bed |  |
|  | Stoker |  |
|  | Rotary kiln |  |
|  | Other (please specify) |  |
| Temperature in Furnace | Main furnace(°C)  Afterburner/second chamber (°C) |  |
| Type of Air Pollution Control | System(APCS)  Electrostatic precipitator  Cyclone  Bag filter  Wet scrubber  Dry scrubber  Lime injection  NaOH/alkali injection  Active carbon/coke injection  Active carbon filter  Catalytic converter (SCR)  Induced or forced draft fan  Other (please specify)  None | [ ]  [ ]  [ ]  [ ]  [ ]  [ ]  [ ]  [ ]  [ ]  [ ]  [ ]  [ ]  [ ]  [ ] |
| **HeatRecoverySystem** |  |  |
| TemperatureofGases | At entry to APCS (°C)[ ] | At exit from APCS(°C) [ ] |
| FluxofExitGases | At exit from APCS(°C) [ ] |  |

Part B: Residue/wasted side-product/bottom ash management

| Residue | Amount | Disposal methods | Landfill disposal & its quantity (t/a) | |
| --- | --- | --- | --- | --- |
| Generation of Bottom Ashes | t/a | Recirculation | Landfill |  |
| Generation of Fly Ashes | t/a | Recirculation | Landfill |  |
| Generation of waste water | t/a | Disposal |  |  |
| Generation of Slides | t/a | Recirculation | Landfill |  |

**Questionnaire3: Group 3 Power Generation and Heating**

**Part A: Power Generation and Heating activity**

| Name of Plant |  | |
| --- | --- | --- |
| Location (City/Province) |  | |
| Address  (Name, position, phone and fax, numbers, email) |  | |
| Type of Plant | **Power plant** | **Response** |
|  | Coal |  |
|  | Lignite |  |
|  | Bituminous coal |  |
|  | Anthracite |  |
|  | Other |  |
|  | Natural gas |  |
|  | Wood |  |
|  | Landfill gas |  |
|  | Sewer gas |  |
|  | Biomass (please specify) |  |
|  | **Industrial Combustion units(small)** |  |
|  | Coal(please specify) |  |
|  | Lignite |  |
|  | Bituminous coal |  |
|  | Anthracite |  |
|  | Other |  |
|  | Natural wood |  |
|  | Combustion of other kindsof biomass |  |
|  | Sugar cane |  |
|  | Tapioka |  |
|  | Cotton |  |
|  | Bamboo |  |
|  | Banana |  |
|  | Harvest residues |  |
|  | Other(please specify) |  |
| Number of Furnaces  Type of Operation | Batch (*e.g.*, 100 kg per batch)  Semi continuous (*e.g.*, 8 hours per day)  Continuous (24 hours per day) |  |
| Annual Operational/Capacity  (per Unit) | t/h(tons per hour) |  |
|  | h/d (hours per day) |  |
|  | d/w (days per week) |  |
|  | t/d (tons per day) |  |
|  | d/a(days per year) |  |
|  | h/a (hours per year) |  |
| Annual Operation/Capacity  (total) | t/a(tons per year)  t/h (tons per hour) |  |
|  | h/d (hours per day) |  |
|  | d/w (days per week) |  |
|  | t/d (tons per day) |  |
|  | d/a (days per year) |  |
|  | h/a (hours per year) |  |
| Type of Furnace | Boiler |  |
|  | Process heater |  |
|  | Flare |  |
|  | Turbine (internal gas) |  |
|  | Combustion engine (internal)  Other (please specify) |  |
| Temperature in Furnace | Main furnace (°C)  Afterburner/second chamber(°C) |  |
| Type of Air Pollution Control | System(APCS) |  |
|  | Electra static precipitator |  |
|  | Cyclone |  |
|  | Bag filter |  |
|  | Wet scrubber |  |
|  | Dry scrubber |  |
|  | Lime injection |  |
|  | NaOH/alkali injection |  |
|  | Active carbon/coke injection |  |
|  | Active carbon filter |  |
|  | Catalytic converter (SCR) |  |
|  | Induced or forced draft fan |  |
|  | Other (pleasespecify) |  |
|  | None |  |
| Temperature of Gases | At entry to APCS (°C) ________ | At exit from APCS(°C) ___ |
| Flux of Exit Gases | (m³/h) (dry gas) |  |

**Part B: Residue management system**

| Residue | Amount | Management methods | Quantity of residue disposed at the Landfill, t/a | |
| --- | --- | --- | --- | --- |
| Generation of Bottom Ashes | t/a | Recirculation | Landfill |  |
| Generation of Fly Ashes | t/a | Recirculation | Landfill |  |
| Generation of Waste Water | t/a | Disposal |  |  |
| Generation of Slides | t/a | Recirculation | Landfill |  |

**Questionnaire 4: Group 4 Production of Mineral Products**

**Part A: Mineral Production**

| Name of Plant |  | |
| --- | --- | --- |
| Location(City/Province) |  | |
| Contact (Name and position, Phone numbers, email) |  | |
| Number of Furnaces  Type of Operation |  | |
| Feed Materials  (type, quantity, t/a) |  | |
| Primary Fuel (type, quantity; t/a) |  | |
| Secondary/Alternative Fuel (type, quantity; t/a) |  | |
| Type of processes |  | |
| Type of operation |  | |
| Type of Plant | **Type** | **Response** |
|  | Lime |  |
|  | Brick |  |
|  | Glass |  |
|  | Ceramics |  |
|  | Asphalt mixing |  |
| Annual Operational/Capacity (per  Unit) | t/a (tons per year) |  |
|  | t/h (tons per hour) |  |
|  | h/d (hours per day) |  |
|  | d/w (days per week) |  |
|  | t/d (tons per day) |  |
|  | d/a (days per year) |  |
|  | h/a (hours per year) |  |
|  | t/a (tons per year) |  |
| Annual Operation/Capacity  (total) | t/a(tons per year) |  |
|  | t/h (tons per hour) |  |
|  | h/d (hours per day) |  |
|  | d/w (days per week) |  |
|  | t/d (tons per day) |  |
|  | d/a (days per year)  h/a (hours per year) |  |
| Type of Furnace | Rotary kiln  Shaft kiln  Tunnel furnace  Other (please specify) |  |
| Temperature in Furnace | Main furnace(°C)  Afterburner/second chamber (°C) |  |
| Type of Air Pollution Control | System(APCS) |  |
|  | Electrostatic precipitator |  |
|  | Cyclone |  |
|  | Bag filter |  |
|  | Wet scrubber |  |
|  | Dry scrubber |  |
|  | Lime injection |  |
|  | NaOH/alkali injection |  |
|  | Active carbon/coke injection |  |
|  | Active carbon filter |  |
|  | Catalytic converter (SCR) |  |
|  | Induced or forced draft fan |  |
|  | Other (please specify) |  |
|  | None |  |
| Heat Recovery System |  |  |
| Temperature of Gases | At entry to APCS (°C)[ ] | At exit from APCS(°C) [ ] |
| FluxofExitGases | At exit from APCS(°C) [ ] |  |

**Part B: Residue management**

| Residue | Quantity | Methods of management | Disposal of these residue and its quantity t/a | |
| --- | --- | --- | --- | --- |
| Generation of Bottom Ashes | t/a | Recirculation | Landfill |  |
| Generation of Fly Ashes | t/a | Recirculation | Landfill |  |
| Generation of Waste Water | t/a | Disposal |  |  |
| Generation of Slides | t/a | Recirculation | Landfill |  |

**Questionnaire 5: Group 5 Transport**

| Location(City/Province) |  |
| --- | --- |
| Address |  |
| Contact  (Name, position, phone and fax, numbers, email) |  |

| Type of Fuel | **Leaded Gasoline** | | **Unleaded Gasoline** | **Diesel/Light Fuel**  **Oil** |
| --- | --- | --- | --- | --- |
| Annual national fuel consumption in liter per year (L/a) |  | |  |  |
| **Passenger Cars** | | | | |
| Number of vehicles |  | |  |  |
| Annual road performance per  vehicle and kilometer (km/a) |  | |  |  |
| Fuelconsumption (L/km;L/a) |  | |  |  |
| Total annual consumption (L/a) |  | |  |  |
| APCS(Yes/No) |  | |  |  |
| **Busses** | | | | |
| Number of busses |  |  | |  |
| Annual road performance per  vehicle and year (km/a) |  |  | |  |
| Fuelconsumption (L/km; L/a) |  |  | |  |
| Totalannualconsumption (L/a) |  |  | |  |
| Annual consumption in tons per  year (t/a) |  |  | |  |
| APCS (Yes/No) |  |  | |  |
| **Busses and Trucks** | | | | |
| Number of busses |  |  | |  |
| Annual road performance per  vehicle and year (km/a) |  |  | |  |
| Fuelconsumption (L/km; L/a) |  |  | |  |
| Total annual consumption (L/a) |  |  | |  |
| Annual consumption in tons per  year (t/a) |  |  | |  |
| APCS (Yes/No) |  |  | |  |
| **Train** | | | | |
| Number of trains (on any of the  above fuels) |  |  | |  |
| Annual railroad performance per  vehicle and year (km/a) |  |  | |  |
| Fuel consumption(L/km;L/a) |  |  | |  |
| Total annual consumption (L/a) |  |  | |  |
| Annual consumption in tons per  year (t/a) |  |  | |  |
| APCS (Yes/No) |  |  | |  |

**Questionnaire 6: Group 6 Opening Burning**

| Name of Plant |  | | | | | |
| --- | --- | --- | --- | --- | --- | --- |
| Location(City/Province) |  | | | | | |
| Address |  | | | | | |
| Contact  (Name, position, phone, fax numbers, email) |  | | | | | |
| **Open Waste Burning and Accidental Fire** | | | | | | |
| General waste statistics |  | |  | |  | |
| Tons of waste generated | Per capita per day | | Per capita and year | | Nationally per year  (t) | |
|  |  | |  | |  | |
| **Type of source** | Amount of waste  Burned per capita (t/a) | | Number of  inhabitants | | Amount of waste  Burned per year (t/a) | |
|  | % | t/a | % | t/a | % | t/a |
| 1. Landfill fire |  |  |  |  |  |  |
| 1. Open burning of domestic waste |  |  |  |  |  |  |
| 1. Open burning of wood (construction/ demolition |  |  |  |  |  |  |
| 1. Accidental fire in houses per year |  | | | |  | |
| 1. Accidental fires in vehicles per year |  | | | |  | |

**Questionnaire 7: Group 7 Production and Use of Chemicals and Consumer Goods Waste Incineration**

**Part A: Production and Use of Chemicals and Consumer Goods Waste Incineration**

| Location(City/Province) |  | |
| --- | --- | --- |
| Address |  | |
| Contact  (Name, position, phone and fax  numbers, email) |  | |
| Capacity: Consumption of Raw  Materials(type, quantity =t/a) |  | |
| Capacity: Final Product of Raw  Materials(type, quantity=t/a) |  | |
| Type of chemical industry/plant | Pulp and paper industry: Pulp |  |
|  | Pulp and paper industry: Paper |  |
|  | (primary or recycling) |  |
|  | Pulp and paper integrated |  |
|  | Organo-chlorine production |  |
|  | Ethylene dichloride |  |
|  | PVC |  |
|  | Pesticides (PCP,2,4,5 T, 2,4 D) |  |
|  | Production of chlorine gas  (graphite electrodes) |  |
|  | Petroleum industry refineries |  |
| Type of Process | Fixed bed |  |
|  | Fluidized bed |  |
|  | Other |  |
| Type of Operation | Batch (*e.g.*, 100 kg per batch)  Semi continuous (*e.g.*, 8 hours per day)  Continuous (24 hours per day) |  |
| Annual Operation/Capacity (per  Unit) | t/h (tons per hour) |  |
|  | h/d (hours per day) |  |
|  | d/w(days per week) |  |
|  | t/d (tons per day) |  |
|  | d/a (days per year) |  |
|  | h/a(hours per year |  |
| Annual Operation/Capacity  (total) | t/h (tons per hour) |  |
|  | h/d (hours per day) |  |
|  | d/w(days per week) |  |
|  | t/d (tons per day) |  |
|  | d/a (days per year) |  |
|  | h/a(hours per year |  |
| Operation/Production  Temperature | System(APCS) |  |
|  | Electrostatic precipitator |  |
|  | Cyclone |  |
| Waterdischarge(L/h,m³/a) | Bag filter |  |
|  | Wet scrubber |  |
| Water Treatment | Settling pond |  |
|  | Aerated lagoon |  |
|  | Secondary treatment |  |
|  | Tertiary treatment |  |
|  | Other (please specify) |  |
| Sludge generation | t/a (tons per year) |  |
| Sludge disposal | Landfill (t/a) |  |
|  | Land farming (t/a) |  |
|  | On site(t/a) |  |
|  | Incineration (t/a) |  |
|  | Other (please specify) (t/a) |  |
| Type of Air Pollution Control  System (APCS)  Electrostatic | System(APCS) |  |
|  | Electrostatic precipitator |  |
|  | Cyclone |  |
|  | Bag filter |  |
|  | Wet scrubber |  |
|  | Dry scrubber |  |
|  | Lime injection |  |
|  | NaOH /alkali injection |  |
|  | Active carbon/coke injection |  |
|  | Active carbon filter |  |
|  | Catalytic converter (SCR) |  |
|  | Induced or forced draft fan |  |
|  | Other (please specify) |  |
|  | None |  |
| Temperature of Gases | At entry to APCS (°C) | At exit from APCS(°C) [ ] |
| Flux of Exit Gases | At exit from APCS(°C) |  |

**Part B: Reside Management**

| Residue | Quantity | Methods of management | Residue disposal and quantity, t/a | |
| --- | --- | --- | --- | --- |
| Generation of Bottom Ashes | t/a | Recirculation | Landfill |  |
| Generation of Fly Ashes | t/a | Recirculation | Landfill |  |
| Generation of Waste Water | t/a | Disposal |  |  |
| Generation of Slides | t/a | Recirculation | Landfill |  |

**Data Collection Tool II: Field Observation Checklist (2017)**

Name of the Organization
Date of Observation

Time of Observations

| S/N | **Issues to be**  **observed** | **Responses from observation** | **Remarks** |
| --- | --- | --- | --- |
| 1 | Are there any policies, laws, or regulations under the organization to manage UPOPs? |  |  |
| 2 | Are there any technology applications and what kinds of technology were used during production? |  |  |
| 3 | What are the existing management practices undertaken by the organizations (combustion and production)? |  |  |
| 4 | What are the management plans or strategies set by the organization to reduce UPOPs? |  |  |

**Data Collection Tool III: Interview guide questionnaire**

1. **Biomedical waste management**
2. How many hospitals, health centers, clinics and other health institutionsare there in Addis Ababa?
3. How many beds are there in all of the health institutions found in the city?
4. How many patients and inpatientsuse the health services? How much biomedical waste is generated?
5. Is there any appropriate legal instrument concerning medical waste incineration?Yes/No
6. If the answer is Yes please mention the legal or policy instrument.
7. Ifthe answer is No, what should be done in the future?
8. What are the technical actions taken by the health centers or clinics during waste combustion?
9. What do you think about the by-products during combustion? Is there any mitigation measure to reduce the effect of those by-products? Pleasemention the mitigation you take.
10. **Ferrous and non-ferrous metal production**
11. How many ferrous and non-ferrous metal production industriesare there in Addis Ababa?
12. How much metallic industries do primary metallurgical processes to obtain metal such as iron, copper, aluminum, lead and zinc?
13. How many metallic industries have primary metallurgical processes that utilize scrap metal, coatedplastics, paints and used batteries?
14. Are there any appropriate legal and policy instrumentsconcerningferrous and non-ferrous metal production? YES/NO
15. If the answer is Yes, please mention the legal or policy instruments.
16. How many industries have automatic air pollution controls?
17. **Power generation and heating**
18. How many power stations, industrial firing places and installations are there in Addis Ababa?
19. How much biomass, such as wood, is used for household heating (tons/annum)?
20. Are there any appropriate legal and policy instruments concerning household heating or biomass burning?YES/NO
21. If your answer is Yes, please mention the legal or policy instruments.
22. **Minerals production**
23. How many brick, asphalt mixing and glass factories are there in the city?
24. What is the total bricks, asphalt mixing and glass produced(tons/annum)?
25. Are there any appropriate legal and policy instruments concerning mineral production? YES/NO
26. If your answer is Yes, please mention the legal or policy instruments.
27. **Transport**
28. How many four stroke enginesand diesel engine cars are there in Addis Ababa?
29. How manyliters of gas imported for four stroke and diesel engine cars per year?
30. Are there any appropriate legal and policy instruments concerning transportation?YES/ NO
31. If your answer is Yes, please mention the legal or policy instruments.
32. **Waste burning and accidental fire**
33. How many tons of solid waste are burned at the landfill site? (tons/year)
34. How many house and factories experience fire in Addis Ababa? (number/year)
35. How much biomass results from the burned houses and factories?

(m^3^/year)

1. **Production and use of chemicals and consumer goods**
2. How many textile and leather industries are there in Addis Ababa?
3. How many tons of textile and leather is produced?
4. Are there any appropriate legal and policy instrumentsconcerning chemical and consumer good production?YES/NO
5. If your answer is Yes, please mention the legal or policy instruments.
6. **Miscellaneous**
7. How many smoke houses and dry cleaning facilities are there in the city?
8. How many cigarettesare smoked in Addis Ababa?
9. How many cigarsare imported into Addis Ababa?
10. **Disposal/landfill**
11. How many sewage treatment plants are there in the city?
12. How much sewage goes through a treatmentplant?
13. Are there any appropriate legal and policy instruments concerning sewage treatment? YES/NO
14. If your answer is Yes, please mention the legal or policy instruments.
15. How many liters of water are openly dumped in the city?
16. How much compost is produced in the city?
