# Supplementary material for: Assessment of Dioxin and Furan Emission Levels and Management Practices in Addis Ababa, Ethiopia
Source: J Health Pollut. 2017 Sep 7;7(15):85–94. doi: 10.5696/2156-9614-7.15.85 (PMC6236542; doi:10.5696/2156-9614-7.15.85)
Supplement: Supplementary file 2 [file Tarakegn_Supplemental_Material_2.doc]

**Supplemental Material 2**

**Respondent Organization Types**

1. **Medical Waste Incineration**

*66 organizations*

1. **Ferrous and non Ferrous**

*2 organizations*

1. **Heat and Power Generation**

*1 organization*

1. **Glass Production**

*2 organizations*

1. **Transport**

*3 organizations*

1. **Open burning**

*2 organizations*

**7. Production and Use of Chemicals and Consumer**

**7.1. Textile Industries**

*6 organizations*

**7.2. Leather industries**

*8 organizations*

1. **Miscellaneous**

**8.1 Dry Cleaning**

*29 organizations*

- 1. **Tobacco Smoking**

*1 organization*

1. **Disposal**

*4 organizations*
